# Crescentic IgA nephropathy along with simultaneous cellular and antibody‐mediated rejection in a kidney transplant leading to rapid allograft failure

**DOI:** 10.1002/ccr3.2364

**Published:** 2019-08-12

**Authors:** Imran Gani, Daniel Kleven, Laura Mulloy

**Affiliations:** ^1^ Department of Nephrology, Hypertension and Transplant Medicine Augusta University Health Augusta GA USA; ^2^ Department of Pathology Augusta University Health Augusta GA USA

**Keywords:** allograft failure, cellular and antibody‐mediated rejection, crescentic IgA nephropathy, immunosuppressant medication noncompliance, kidney transplant

## Abstract

Crescentic IgA Nephropathy in a renal transplant can lead to rapid loss of graft function despite treatment. Concurrent treatment‐resistant acute cellular and antibody‐mediated rejection make the prognosis even worse.

## 1 INTRODUCTION

De novo or recurrent IgA nephropathy can occur in a kidney transplant. Also, transplant rejection is a common cause of allograft dysfunction in patients with kidney transplant. Rejection can be either cellular or antibody‐mediated and the treatment options differ for both. Crescent formation is commonly seen in severe glomerular injury and can cause rapid loss of renal function. We report a unique case of acute renal failure in a kidney transplant patient secondary to crescentic IgA nephropathy along with simultaneous acute cellular and antibody‐mediated allograft rejection resulting in rapid allograft failure.

## 2 CASE REPORT

**Figure 1:**
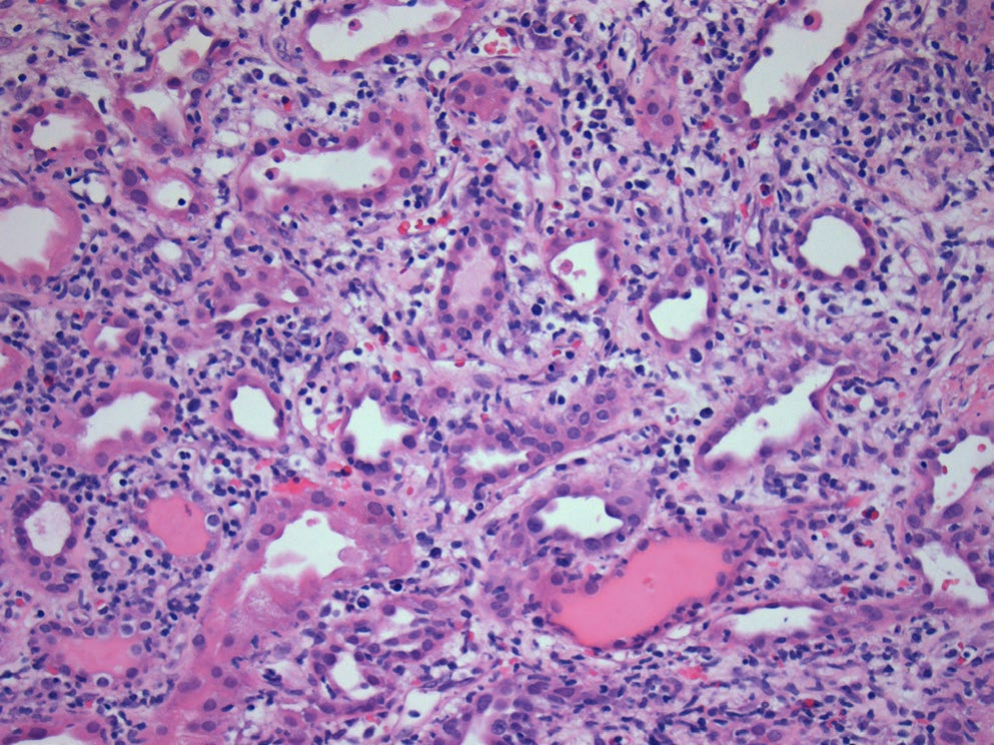
Severe tubulointerstitial infiltrate with numerous tubules showing tubulitis. (Hematoxylin and Eosin 200×)

**Figure 2:**
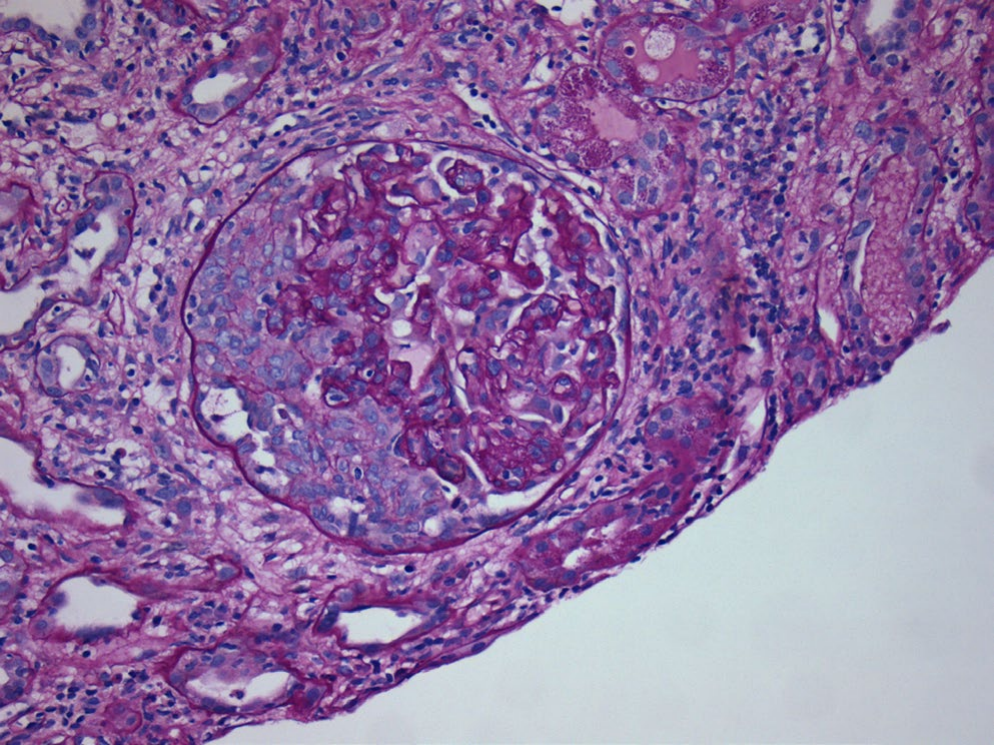
Glomerular Crescent. The capillaries also show mononuclear cells indicative of glomerulitis. (Periodic Acid Shiff 200×)

**Figure 3:**
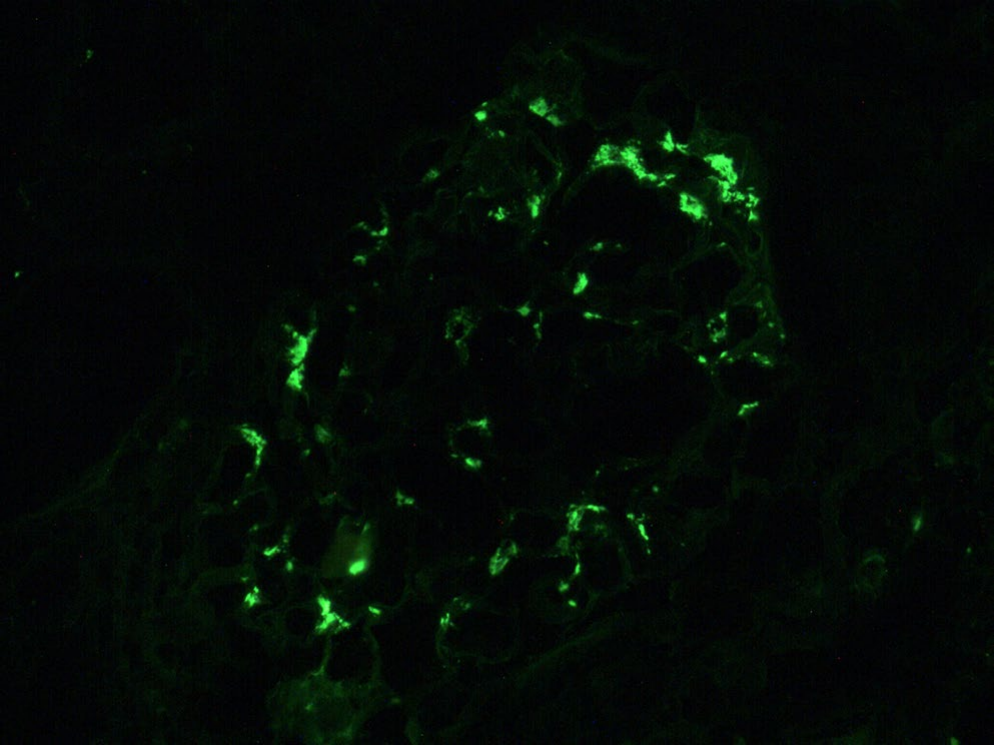
IgA immunofluorescence showing mesangial deposition of IgA. (Anti‐IgA fluorescein‐labeled antibodies 200×)

A 36‐year‐old Caucasian man developed end‐stage renal disease (ESRD) secondary to recurrent acute kidney injury (from recurrent sepsis due to chronic hip methicillin‐resistant staphylococcus aureus osteomyelitis) and contrast exposure. He was on dialysis for 9 years before receiving a deceased donor kidney transplant in February 2015. He had an episode of combined cellular and antibody‐mediated rejection in October 2015 due to noncompliance with his immunosuppressive medications which was successfully treated with pulse steroids, plasmapheresis, intravenous immunoglobulin, and rituximab. After that, his baseline creatinine was around 1.5‐1.7, last checked in December 2015. He did not follow‐up until November 2017 with a creatinine of 2.3. In January 2018, he was admitted with severe nausea, dark‐colored urine, and diarrhea. On physical examination, he had elevated blood pressure and 1+ bilateral lower extremity edema. There was no skin rash. Laboratory evaluation revealed acute kidney injury with a creatinine of 11 mg/dL (new baseline 2.3), BUN of 96 mg/dL, metabolic acidosis with bicarbonate of 11 mEq/L, potassium of 5.6 mEq/L, and subtherapeutic tacrolimus level of 1.6 ng/mL. Urinalysis and microscopy revealed dysmorphic RBCs and proteinuria without any evidence of infection. Patient admitted to being noncompliant with his immunosuppressant medication. Transplant ultrasound did not show any obstruction or vascular compromise. Due to worsening acidosis and hyperkalemia, hemodialysis was initiated. Transplant kidney biopsy was performed which revealed mesangial expansion, glomerular crescents (in 75% of the glomeruli on light microscopy sample), severe lymphoplasmacytic tubulointerstitial infiltration, glomerulitis, infiltration of the peritubular capillaries by inflammatory cells (peritubular capillaritis), mononuclear infiltrate along arterial intima (transplant arteritis), and subendothelial expansion and duplication along the glomerular basement membranes (transplant glomerulopathy; Figures 1, 2). Immunofluorescence showed mesangial IgA (Figure 3) and C3 deposition, positive C4d along peritubular capillaries. IgM, IgG, and C1q immunofluorescent stainings were negative. Ultrastructural evaluation revealed mesangial immune complex deposits associated with mesangial expansion. Immunohistochemical staining for SV 40 (BK Virus) was negative. The biopsy findings were consistent with crescentic IgA nephropathy along with both cell mediated as well as humoral rejection in the transplanted kidney. Donor‐specific antibodies were positive in very high titers in our patient. Further glomerulonephritis workup revealed positive antinuclear antibodies, mildly depressed C3, normal C4 level and negative C‐ANCA, P‐ANCA, anti‐GBM, cryoglobulins, HIV, hepatitis panel, and RPR test. Blood cultures, urine culture, EBV, CMV, adenovirus, BK virus, and influenza virus testing were all negative. There were no vegetations on echocardiogram.

Treatment was initiated with high‐dose pulse steroids, thymoglobulin, intravenous immunoglobulin, and seven sessions of plasma exchange. Cyclophosphamide was not used. Despite these measures, the patient never recovered good allograft function and ended up on maintenance hemodialysis.

## 3 DISCUSSION

Glomerulonephritis seen in renal allografts is typically the recurrences of the disease affecting the native kidneys. Occasionally, however, a new‐onset of a different type of glomerulonephritis can develop in the transplanted kidney.^1^ The de novo and recurrent glomerular disease may have same clinical and histological features, although the unique set of pathologies unique to the kidney allograft such as cellular and antibody‐mediated rejection, calcineurin inhibitor nephrotoxicity, viral infections may make the diagnosis challenging.^2^

IgA nephropathy can present in a kidney transplant as a recurrent disease in a patient with a known history of IgA nephropathy in the native kidneys or as a new‐onset IgA nephropathy termed as de novo IgA nephropathy. IgA nephropathy may also manifest after a patient has received a kidney transplant that already had a “latent” IgA nephropathy. Not all allografts are biopsied before transplantation. Suzuki et al, reported mesangial IgA deposition present in 16.1% of kidney allografts that were biopsied at zero hour (at the time of transplantation).^3^ Mesangial IgA deposits can be clinically silent and are diagnosed on protocol biopsies or on biopsies done for other clinical reasons, such as elevated creatinine or proteinuria. In a study comparing donor kidneys with glomerular mesangial proliferation and marked diffuse granular IgA deposits, the recipients had a higher incidence of delayed graft function and acute rejection as compared with donor kidneys without such deposits. Long‐term allograft survival, however, was not significantly different.^4^ Both recurrent and de novo IgA nephropathy can lead to renal allograft dysfunction. IgA nephropathy associated with glomerular crescent formation, whether recurrent or de novo, can cause accelerated allograft dysfunction which may not be fully reversible with treatment.

A study from Korea reported lower allograft survival and chronic graft dysfunction in crescentic post‐transplant IgA nephropathy.^5^ In a study by Tang et al, 10 kidney transplant patients were diagnosed with recurrent or de novo IgA nephropathy with crescent formation (mean crescents 37.5%), all patients had progressive renal dysfunction, nine returned to hemodialysis after 6‐36 months, with only one patient whose renal function remained stable with a creatinine of 2.5 mg/dL for 3 years at follow‐up.^6^ Shabaka et al presented a case of de novo IgA nephropathy after renal transplant presenting with progressive deterioration of renal function which responded to treatment, with gradual improvement in renal function and proteinuria, yet microscopic hematuria persisted.^7^ De novo IgA nephropathy with the presence of crescents usually has an aggressive presentation which can lead to loss of allograft. This form of the disease may occur at any time after transplantation. In a study by Kowalewska et al, recurrent or de novo crescentic IgA nephropathy led to return to dialysis therapy in half of the affected patients.^8^

Our patient's biopsy done in October 2015 at another hospital was read as acute cellular rejection, acute vascular rejection, moderate microcirculatory inflammation with C4d positivity, highly suspicious of antibody‐mediated rejection. Nonspecific IgM and IgA staining along peripheral capillary loops and mesangium, consistent with trapping in areas of reduplication of glomerular basement membrane, and areas of mesangiolysis were seen. There were no mesangial immune complex deposits by electron microscopy. There were no crescents. The sister donor kidney was traced, although it has never been biopsied post‐transplant, the recipient has a normal creatinine with no hematuria as of November 2018. With no past medical history of IgA nephropathy in our patient, his first kidney biopsy not considered as IgA nephropathy and a normal creatinine with no hematuria in the sister kidney, de novo IgA nephropathy is a more favorable diagnosis in our case.

Transplant rejection is a major cause of renal transplant dysfunction and loss. In some cases, renal transplant dysfunction due to rejection is irreversible even with maximal antirejection therapy. Transplant rejection usually manifests as an elevation in serum creatinine. Patients may also have hematuria, graft tenderness, and fever. Diagnosis is made by kidney transplant biopsy. Acute cellular rejection is characterized by infiltration of the interstitium and tubules with mononuclear cells. In cell‐mediated vascular rejection, lymphocytes and monocytes undermine arterial endothelium causing arteritis which can be transmural and associated with arterial fibrinoid change and necrosis of smooth muscle cells.^9^ Chronic cellular rejection can have arterial intimal fibrosis with mononuclear cell infiltration in fibrosis and formation of neo‐intima. Antibody‐mediated rejection is associated with characteristic histological changes, glomerulitis, peritubular capillaritis, and in most cases with C4d‐positive staining and presence of donor‐specific antibodies. Chronic antibody‐mediated rejection is associated with transplant glomerulopathy and peritubular capillary basement membrane multi‐layering.^10^

Kidney transplant patients need lifelong follow‐up and maintenance on immunosuppressive medications. Adherence to immunosuppressant medications in the post‐transplant period is an important determinant of both short‐term and long‐term graft outcomes. Immunosuppressant nonadherence is a risk factor for late acute as well as chronic rejection and allograft failure.^11^ The reported prevalence of immunosuppressive medication nonadherence in kidney transplantation recipients varies greatly, from as low as 2% to a very high of 65%, depending on the definition of prevalence used, the methods used for its measurement and the transplant population studied.^12^ A weighted mean prevalence of nonadherence was 27.7% when measured by self‐report in a literature review.^12^ A meta‐analysis showed that immunosuppressive medication nonadherence was highest in kidney recipients with 35.6 cases per 100 persons per year.^13^

Our case reports occurrence of crescentic IgA nephropathy in conjunction with both cellular and antibody‐mediated rejection in a kidney transplant patient who was not compliant with his immunosuppressant medications. IgA nephropathy with mesangial deposits alone generally has a favorable prognosis but presence of crescents heralds a bad prognosis. Compounding the renal transplant injury in our patient was the simultaneous acute cellular and antibody‐mediated rejection, making recovery of renal function most difficult. Various treatment regimens including steroids, cyclophosphamide, plasma exchange have been used for the treatment of post‐transplant recurrent crescentic IgA nephropathy. Our patient did not respond to the treatment regimen consisting of high‐dose pulse steroids, thymoglobulin, plasma exchange, and intravenous immunoglobulin. Kidney Disease Improving Global Outcomes (KDIGO) guidelines suggest steroids and cyclophosphamide for crescentic IgAN with rapidly deteriorating kidney function in native kidneys.^14^ Although its use has been reported, cyclophosphamide is not an established treatment of crescentic IgA nephropathy recurrence in a kidney transplant. Diaz‐Tejeiro R et al reported a rapidly progressive recurrent crescentic IgA nephropathy unresponsive to treatment with cyclophosphamide, plasma exchange, and steroids leading to return to hemodialysis.^15^ Robles et al reported a post‐transplant crescentic IgA nephropathy with a rapidly progressive course that did not achieve long‐term remission with cyclophosphamide, plasmapheresis, and steroid treatment.^16^ There are, however, reports of successful treatment of recurrent post‐transplant crescentic IgA nephropathy with cyclophosphamide‐containing regimen. Zagkotsis et al reported successful treatment and long‐term remission in a patient with recurrent post‐transplant IgA nephropathy treated with steroids and cyclophosphamide.^17^ Gopalakrishnan et al report a good response to pulse steroids and intravenous cyclophosphamide in a case of recurrent crescentic IgA nephropathy in which 56% of glomeruli showed crescent formation.^18^ We chose not to use cyclophosphamide in our case due to the high likelihood of nonreversibility of kidney transplant dysfunction given the extent of pathologic findings, as well as concern for excessive immunosuppression.

## 4 CONCLUSION

Crescentic IgA nephropathy may have a rapidly progressive clinical course with a poor functional outcome in renal transplant recipients. Simultaneous acute cellular and antibody‐mediated rejection may sometimes not respond to treatment as well. In our case, the presence of both of these disease processes culminated in the rapid loss of allograft function despite a robust treatment regimen.

## CONFLICT OF INTEREST

None.

## AUTHOR CONTRIBUTIONS

Imran Gani: case report and discussion compilation, review of literature, management of the patient. Daniel Kleven: reviewed the renal transplant biopsy and provided the images. Laura Mulloy: reviewed the case report and was involved in the management of the patient.
